# Diabetes in rural towns: effectiveness of continuing education and feedback for healthcare providers in altering diabetes outcomes at a population level: protocol for a cluster randomised controlled trial

**DOI:** 10.1186/1748-5908-8-30

**Published:** 2013-03-13

**Authors:** Christine L Paul, Leon Piterman, Jonathan Shaw, Catherine Kirby, Robert W Sanson-Fisher, Mariko L Carey, Jennifer Robinson, Patrick McElduff, Isaraporn Thepwongsa

**Affiliations:** 1 Health Behaviour Research group, Priority Research Centre for Health Behaviour, School of Medicine and Public Health, University of Newcastle, HMRI Building, Callaghan, NSW 2308, Australia; 2 The Hunter Medical Research Institute, Callaghan, NSW 2308, Australia; 3 Office of the Pro Vice-Chancellor (Berwick and Peninsula), Monash University, Building 901, 100 Clyde Road, Berwick, VIC 3806, Australia; 4 Baker IDI Heart and Diabetes Institute, PO Box 6492St Kilda Road Central, Melbourne, VIC 8008, Australia

**Keywords:** Type 2 diabetes mellitus, Cluster randomised controlled trial, Internet, Medical education, General practitioner

## Abstract

**Background:**

Type 2 diabetes is one of the fastest growing chronic diseases internationally. The health complications associated with type 2 diabetes can be prevented, delayed, or improved via early diagnosis and effective management. This research aims to examine the impact of a primarily web-based educational intervention on the diabetes care provided by general practitioners (GPs) in rural areas, and subsequent patient outcomes. A population-level approach to outcome assessment is used, via whole-town de-identified pathology records.

**Methods/design:**

The study uses a cluster randomised controlled trial with rural communities as the unit of analysis. Towns from four Australian states were selected and matched on factors including rurality, population size, proportion of the population who were Indigenous Australians, and socio-economic status. Eleven pairs of towns from two states were suitable for the trial, and one town from each pair was randomised to the experimental group. GPs in the towns allocated to the experimental group are offered an intervention package comprising education on best practice diabetes care via an on-line active learning module, a moderated discussion forum, access to targeted and specialist advice through an on-line request form, and town-based performance feedback on diabetes monitoring and outcomes. The package is offered via repeated direct mail.

**Discussion:**

The benefits of the outcomes of the trial are described along with the challenges and limitations associated with the methodology.

**Trial registration:**

Australian New Zealand Clinical Trials Registry: ACTRN12611000553976

## Background

Type 2 diabetes is one of the fastest growing chronic diseases internationally [1]. It is estimated that world prevalence of diabetes in adults will rise from 6.4% in 2010 to 7.7% by 2030 [2]. Long-term complications associated with diabetes include macrovascular disease, coronary heart disease, stroke, retinopathy, and kidney diseases [3]. Those diagnosed with diabetes have double the risk of developing cardiovascular disease (CVD) and have a higher mortality due to their first CVD event [3,4]. Diabetic nephropathy affects approximately 10% of people with diabetes, is the most common cause of end-stage kidney disease, and diabetes is a major contributor to lower limb amputations and visual loss [3]. The direct and indirect costs of diabetes and its associated complications to the health system and to individuals is substantial [5].

The health complications associated with type 2 diabetes can be prevented, delayed, or lessened if diabetes is diagnosed early and is well controlled. While there are many factors important to diabetes control, metabolic markers of poor diabetes control such as elevated blood glucose, blood lipids, cholesterol, and urinary albumin indicate increased risk of diabetic complications [6-11]. Interventions designed to impact on multiple risk factors in people with diabetes can reduce risk of diabetes complications [6,12]. However, achieving optimal diabetes management at a population-level remains challenging.

General practitioners (GPs), or primary care physicians, play a major role in diabetes management [3]. In an effort to improve diabetes management, national Clinical Practice Guidelines for general practice specify the requirements for testing of blood lipids, HbA1c, and urinary albumin [6,13]. Target levels for each of these metabolic indicators are provided, and follow-up care is recommended if results fall outside these parameters [13]. While these metabolic markers cannot assess the quality of an individual’s diabetes care, they represent key objective measures of the management of diabetes at a population level. While there were about 818,000 Australians diagnosed with diabetes in 2009 and 2010, it appears that a minority have received the recommended annual testing of metabolic markers [14].

In Australia, people living in regional, rural, and remote regions have mortality rates between 10% and 70% higher than those living in the major cities [15]. Rural and remote communities are of particular importance because rates of diabetes consultations are higher than in other areas of Australia [16]. Rates of diabetes-related hospitalisation rise with increasing remoteness of residence [3], as do death rates due to diabetes [3]. The proportion of diabetic patients meeting targets for total cholesterol, triglycerides, and blood pressure levels is also lower in rural areas compared with urban areas [17]. The role of the GP is critical in rural settings, because there may be less access to specialist services. In Australia, organisations such as the Rural Health Education Foundation run distance education programs, including internet-based programs for rural GPs; however, these have not been evaluated for effect on quality of care or patient outcomes.

Continuing medical education (CME) is a commonly employed mechanism to improve clinical practice [17]. There is growing evidence that CME approaches that involve multiple exposures to educational material over time and a variety of educational techniques are effective at improving doctors’ knowledge, attitudes, and patient outcomes [18]. Web-based CME is increasing in popularity and is of particular relevance to GPs in non-metropolitan locations where face-to-face training opportunities are less accessible. However, while some studies have examined the effect of CME on participant satisfaction [19], few have examined the effect on patient outcomes, and none at the population level.

Studies that examine the effect of provider-change strategies on patient outcomes have done so by looking at patients linked to providers who have participated in the study [20-22]. The outcomes at the patient level, therefore, do not include the patients of providers who choose not to participate, patients who are ‘missed’ or excluded by their provider, patients who do not provide consent, and providers or patients lost to follow-up. While these methodological flaws are often unavoidable, they severely limit the generalisability of the results. This project will use objective data to examine uptake and effectiveness of CME and additional intervention strategies at the population level in the rural setting. To meet this objective, the research design uses communities as the unit of analysis and administrative data sets (whole town de-identified pathology data) without the usual problems of generalisability resulting from enrolling practices and patients.

In addition to drawing on best evidence for CME [18] the intervention will be multifaceted; including an on-line Active Learning Module (ALM) attracting CME points, and strategies designed to improve adherence to guideline recommendations such as performance feedback [23,24]. Non-metropolitan GPs are likely to be receptive to the web-based aspects of our proposed approach because computer use by GPs in rural and remote areas is higher than that of GPs in metropolitan areas [16]. Rural towns also offer a greater chance of capturing the whole population than does a selected group of GPs.

### Aims and objectives

#### Objective

To test whether population-level improvements can be achieved by implementing evidence-based practice change strategies for GP care and management of diabetes in rural communities.

#### Primary aim

To test whether a rural GP-focused intervention involving Continuing Medical Education (CME), reminders and feedback can improve clinical outcomes as measured by glycaemic control.

#### Secondary aims

To examine whether the above-mentioned intervention can:

1. Improve patterns of diabetes care as measured by whether the frequency of testing for hemoglobin A1c, blood lipids and urinary albumin meets NHMRC (National Health and Medical Research Council) guidelines.

2. Improve other clinical outcomes as measured by blood lipid control and urinary albumin control.

## Method/design

### Study design

The study uses a cluster randomised controlled trial with towns as the unit of allocation and analysis. This enables the impact of the interventions to be examined at a population level and allowed the outcomes related to diabetes care for individuals to be examined regardless of whether care was sought from one or multiple GPs within their community. Randomisation by town may result in similar intervention effects even if patients change GPs within their town over the course of the study. Towns were randomised via a computer-generated stratified randomisation scheme in SAS (Statistical Analysis Software). Allocation remained concealed to all participants and all those assessing outcomes throughout the study. Because the clusters were towns, consent was not required from clusters or members of clusters. Management of the various challenges associated with this design is addressed in the discussion.

### Selection and matching of town sample

Towns were defined by postal area and matched to town names through the Australia Post website. Towns were eligible for selection if they: had an ‘Australian Remoteness Index for Areas Plus’ (ARIA+) [25] classification of 2.0 or greater; had a population of 10,000 to 30,000 people; were in the Australian states of Victoria, New South Wales (NSW), or Queensland, *i.e.*, where the two collaborating pathology companies operate with good coverage of regional and rural towns; and had five or more full-time equivalent GPs according to the Medical Directory of Australia and cross-matched with census data. This criterion was included to ensure GP and patient anonymity. This process identified a possible 43 towns.

Towns were matched in pairs within each state on the above variables, proportion of population identified as Indigenous, and socio-economic status (SES). Indigenous population was incorporated in the matching process because the prevalence of diabetes is higher among Indigenous Australians than in the general population [26]. SES using the Socio-Economic Index for Areas (SEIFA) was also included in the matching process because diabetes was two to two-and-a-half times more prevalent among those in the lowest versus the highest socioeconomic group [26]. The town matching process was as follows: Towns were sorted by total population, and pairs selected within each state which were the most similar; priority was given to average ARIA+, then total population, followed by Indigenous population (although for instances of a large difference in indigenous population between two towns, general population was of less importance), and then SEIFA, because all towns had similar SEIFA codes. Once towns had been divided into pairs (NSW = 7 pairs, Queensland = 6 pairs, Victoria = 2 pairs), they were ranked according to the ‘best’ pairs. A minimum distance of 100 km between paired towns was required to prevent contamination between experimental and control communities. An initial matching process incorporating all of the above criteria for inclusion, exclusion, matching, and contamination prevention indicated that there were 17 potentially matched pairs of towns (*i.e.*, 34 towns). The pathology laboratories operating in each of the 34 towns were verified via discussion with pathology providers and searching of the electronic yellow pages to identify any towns where the two collaborating pathology laboratories provided a low proportion of the pathology services. This excluded a further six pairs of towns, leaving 11 matched pairs of towns, all of which were in NSW and Queensland. One town from each matched pair was randomly allocated to the intervention group and the other to the control group. As indicated in the sample size section, a minimum of seven matched pairs (14 towns) will be required at follow-up. Given the possibility that pathology laboratory presence in a selected town may change over the course of the study with the result that one or more towns may become ineligible, all 11 town pairs were included in the study.

### Selection of case sample

For the two-year baseline period, the two pathology companies used the postcode of the treating doctor to identify the potential cases in each study town. The existence of at least one HbA1c test was the criterion for an individual to be identified as a potential case of diabetes. A study case was defined by having either a single HbA1c test where the result was greater than 7%, or two HbA1c tests within the study period. This approach was used to exclude those cases where a single HbA1c test had been used to screen for diabetes. This criterion may have resulted in the omission of some cases of diabetes (*i.e.*, people with diabetes who had not had an HbA1c test). However, more serious forms of bias are avoided, because neither provider consent nor patient consent was required.

### Data extraction procedure

For each case the following de-identified unit record data were extracted from pathology laboratory records: date of birth, gender, de-identified doctor code, postcode of doctor’s clinic, postcode of patient, date and value of each HbA1c test, date and value of each cholesterol test, date and value of each triglycerides test, date of each HDL cholesterol test, date and value of each LDL cholesterol test, and date of each urinary albumin test. The unit record data were extracted for three time periods: pre-test measures were taken over the 24 months prior to the intervention retrospectively; during the intervention, measures were collected prospectively; post-test measures will be taken in the 24 months following the termination of the intervention. Where both pathology companies operate in the same town, the two separate data sets will be combined to produce a single data set for each town.

### Measures

#### Primary outcome

##### Glycaemic control

The incidence and progression of complications of diabetes are reduced for people with lower HbA1c [8-10]. Therefore, guidelines recommend an overall target of ≤7.0% for HbA1c [6]. Using the most recent HbA1c for each case within each 24-month timeframe, the mean HbA1c across the town, and the proportion of all cases whose most recent HbA1c value is ≤7.0%, was determined.

#### Secondary outcome

##### Frequency of HbA1c testing

NHMRC guidelines [6] recommended HbA1c testing every six months if the result was <7.0%, with re-testing to occur within three months if the result was ≥7.0%. Over each 24-month period, the number of all cases where there was at least one gap between tests of more than six months (seven months or greater) was determined and aggregated for each town. For each case with at least one HbA1c test >7.0%, the number of cases where a subsequent gap between HbA1c tests of more than three months (four months or greater) was determined and aggregated for each town. The mean gap between HbA1c tests for each case was also determined.

##### Frequency of blood lipid and urinary albumin testing

Clinical practice guidelines recommended that blood lipids and urinary albumin levels were tested once every 12 months as part of an annual cycle of care, with re-testing to occur within three months if the result was outside recommended levels [13]. Over each 24-month period, the number of all cases where there was at least one gap between tests of more than 12 months (13 months or greater) was determined and aggregated for each town. For each case with at least one test outside recommended target levels [13], the number of cases where a subsequent gap between tests of more than three months (four months or greater) was determined and aggregated for each town. The mean gap between blood lipid and urinary albumin tests for each case was also determined.

##### Blood lipids and urinary albumin control

The following targets were recommended for blood lipids: total cholesterol <4.0mmol/L; triglycerides <1.5mmol/ L; HDL cholesterol >1.0 mmol/L; LDL cholesterol <2.5mmol/ L [13]. A urinary albumin excretion of <20 μg/min is considered normal [13]. Using the most recent results for each case within each 24-month timeframe, the mean values of blood lipids and urinary albumin and the proportion of all cases whose most recent values are within the relevant target ranges was determined.

##### Process measures

The uptake and implementation of the intervention were monitored in terms of: number of GPs who commence and complete the on-line ALM (described below); number and type of interactions on the discussion forum; and number and content of requests to the diabetes specialist via the on-line request form (described below). GPs who registered for the ALM were also invited to complete on-line surveys at pre-test and post-test regarding self-reported knowledge, attitudes, awareness of need for change (pre-test), intended changes in practice (immediate post-test) or actual changes in practice (follow-up). The surveys consisted of multiple choice, yes-no, and open-ended questions.

##### Intervention group

Systematic reviews identified several CME approaches that were more effective than others. These included: use of multimedia rather than single media; use of multiple educational techniques (*e.g.*, case based learning, discussion groups, personal reflection or audit); more than one exposure to the educational material [18]; and opportunities for practice [27]. GPs in towns allocated to this group were offered an intervention package comprising several features: CME on best practice diabetes care via an on-line ALM, including a moderated discussion forum, self-audit and pre-post ALM reflection exercise, access to specialist advice through an on-line request form, and town-based performance feedback on diabetes monitoring and outcomes. The package was offered via repeated direct mail using evidence-based strategies, including primer postcards, personalised mail, CME or quality improvement (QI) and Continuing Professional Development (CPD) points, reminder gifts (jellybeans and tent calendars with reminder messages), faxed reminders, and an incentive of $200 for ALM completion [28-32]. The content addressed the full range of best-practice care, not solely monitoring metabolic markers, was pilot-tested and drew on the expertise of the Monash Department of General Practice and the Baker IDI Heart and Diabetes Institute. The intervention is to be conducted over two years and designed to not only provide GPs with prerequisite knowledge for optimal primary care management of diabetes, but to provide opportunities to practice and refine skills in the practice setting. The intervention components are:

1. On-line active learning module (ALM): This component of the intervention targeted GPs’ knowledge of diabetes care as a necessary but not sufficient factor for practice change. Because multimedia education is more effective than a single medium [18], the ALM included a range of features and presentation types including: evidence-based Australian clinical guidelines, video demonstrations, case studies, knowledge-based quizzes, clinical audit, self-reflection activities, and a moderated peer discussion forum. The ALM comprised approximately six hours of learning activity, for which GPs received CPD points via their professional body. Content for the ALM was developed in consultation with rural GPs and diabetes specialists to ensure accuracy and relevance to the rural GP population. The online ALM was trialled by a small sample of GPs and diabetes specialists to ensure that the final program was user-friendly, accurate, and relevant to GPs.

2. Access to specialist advice: Because rural GPs may not have access to a diabetes specialist in their town, every mailing to promote the ALM also promoted the availability of an on-line request form for specialist advice regarding diabetes, accessed by secure log in. A diabetes specialist was assigned to manage queries from GPs in the intervention towns. This enabled GPs to access advice on applying the diabetes management principles learned in the educational program in their day-to-day practice, and to ask for advice on more complex cases that arose. Importantly, this represents a mechanism for case-based learning, which has been demonstrated to be one of the most promising forms of CME [18].

3. Provision of town-based performance feedback: GPs were provided with town-based feedback which contained de-identified information about the proportion of diabetic patients in the town receiving testing at the recommended frequency and within target guidelines for HbA1c and blood lipids. Systematic reviews show that audit and feedback can have modest effects on changing provider practice [24].

4. Provision of comparative feedback: GPs were provided with a summary of the range of performance per GP in their town. This takes the form of a graph showing the proportion of patients per GP who meet guidelines for test frequency and management (meeting HbA1c targets). Comparative feedback has been shown to be an effective means of changing surgeons’ behavior [33,34]. Both forms of feedback were derived from de-identified data provided by local pathology laboratories.

##### Control group

No interventions by the research team are being provided to this group. There ae no restrictions on GPs’ access to other available forms of diabetes information and education opportunities during the period of the study.

##### Sample size

Assuming that 50% of subjects will be controlled in the usual-care group (*i.e.*, have a HbA1c level below 7%) and that an absolute increase of 10% is important (*i.e.*, an increase to 60% in the intervention group) then, assuming no cluster effect, the study would require 408 subjects per treatment arm to have 80% power of detecting a 10% difference at the 5% significance level. However with 14 clusters, an average of 464 diabetic patients within each cluster and an estimated intraclass correlation associated with the outcome of 0.015, the design effect of this study will be 7.9, and therefore a total of 6,490 subjects will be required. Assuming each of the 14 towns has an average of 20,000 persons and an average rate of being treated for diabetes of 2.9%, there would a total of 8,120 subjects with diabetes in the study.

##### Statistical methods

Changes in the proportion of patients in each town exhibiting glycaemic control, appropriate frequency of testing for each of HbA1c, blood lipids, urinary albumin, and control of lipids and albumin will be assessed at baseline and post-test as described in the measures section. Analysis of the predictors of metabolic control, using age and gender will also be assessed. Analyses using cluster-level summaries are more robust than analyses based on individual-level data when there are fewer than 15 clusters per treatment arm [35]. Therefore, the primary outcome for this study will be analysed by calculating the proportion of subjects with a HbA1c level below 7% within each cluster and then comparing the proportion of the intervention group with the proportion of the control group using a two sample t-test. The main analysis will be conducted with all the available data using the intention-to-treat principle. Subgroup analyses will be conducted by gender and age group. A similar approach will be used to test the secondary outcomes.

##### Trial status

The study has ethical approval from the Human Research Ethics Committees of Monash University and the University of Newcastle. The trial has been registered as a trial at the Australian New Zealand Clinical Trials Registry (ANZCTR) registration number ACTRN 12611000553976 at http://www.ANZCTR.org.au/ACTRN12611000553976.aspx. To date, the baseline measurement phase of the trial has been completed and the intervention phase is nearing completion.

## Discussion

The study methodology will deliver a community-level estimate of the effectiveness of a practice-change intervention for diabetes, providing valuable information regarding the implementation of large-scale strategies for practice change.

### Challenges and limitations

There were a number of challenges to consider in the execution and analysis of this type of study. Below is a description of some of the major challenges and how they were managed.

### Definition of a type 2 diabetes ‘case’

A study case was defined as having either a single HbA1c test where the result was greater than 7%, or two HbA1c tests within the study period. This approach was used to exclude those cases where a single HbA1c test had been used to screen for diabetes. While this criterion resulted in the omission of some cases of diabetes (*i.e.*, those who had not had an HbA1c test), the scale of the study and avoidance of participant bias was considered to compensate for any potential impact on generalizability of results. Some individuals may travel, receiving their care from specialists outside the selected communities. However, any such effect is likely to be similar across experimental and control communities.

### Use of the HbA1c test as a screening tool

The HbA1c test was not recommended as a screening test in Australia at the time of study commencement and does not attract Medicare reimbursement when used as a diagnostic test. Individuals with only one HbA1c test where the test result is below 7% will be excluded from the sample. Therefore, should the use of the HbA1c test as a screening tool have increased during the study period, it is unlikely to influence the study outcome. Should such a change occur, it will be possible to assess using interrupted time series analysis whether an overall increase in the use of one-off HbA1c tests has occurred over time, because the study data on HbA1c testing will provide a near-complete record of all HbA1c testing in the study communities over the five-year study period.

### Variation in pathology assay techniques and instrumentation

HbA1c analysis results are subject to variation as a result of the type of equipment used, with the analysis method accounting for differences of up to 23% in the result [36,37]. In the course of this study, changes in the equipment and methods used for HbA1c analysis were monitored via communication with the two pathology companies to assess the need for sensitivity analyses exploring the size of any such effect.

### Variation between guidelines on HbA1c test frequency

Both the NHMRC and Royal Australian College of General Practitioners (RACGP) have guidelines on diabetes management. The NHMRC is the national body that provides authoritative, evidence-based national guidelines on a wide range of health matters. The RACGP guidelines are designed specifically for the general practice setting and are available in a handbook along with a full range of matters relevant to general practice. The NHMRC guidelines stated that HbA1c testing should occur every six months [38]. While the RACGP guidelines nominated six-month testing of HbA1c, the document had a focus on the 12-month cycle of care and the associated government funding for annual testing [13]. While the ALM and the feedback to GPs focused on the six-month interval, it is possible that GPs may have placed more weight on the 12-month interval. Therefore, analyses will address both six-month and 12-month intervals for HbA1c testing.

### Pathology testing occurring outside the participating pathology companies or towns

Although considerable efforts have been made to include only towns where the two participating pathology companies provided the vast majority of pathology services, it is possible that in some towns a small proportion of pathology testing may have occurred at other locations. For example, some pathology testing may have been sent to hospitals in larger towns nearby. At regular intervals during the course of the study, a sample of GPs were asked by telephone where they sent their pathology in order to assess whether this was occurring. It was also possible that individuals may on occasion have travelled to other towns for testing.

### Matching and merging records for de-identified patient data across pathology companies

The two pathology companies independently provided de-identified unit record data for each ‘case’. Therefore, the data was obtained as two independent data sets that were merged to provide a profile for all individuals in each town. Gender and date of birth were used to identify any individual who may occur in both datasets and data was merged for that individual.

### Individuals may move into or out of the dataset over time due to death, geographical re-location, or initial diagnosis

The data were analysed on the assumption that individuals entering or leaving a town would occur at a reasonably constant rate in each town. These effects would be expected in equal proportions across the intervention and control towns.

### Standard population guidelines may not be appropriate for all cases

Higher cut-off points for glycaemic control (*e.g.*, 8% for HbA1c in older patients) may be recommended in some cases. The primary outcome analysis relates to changes in mean HcA1c levels and would, therefore, be relatively unaffected by this factor. Analyses relating to the proportion of cases in each town achieving glycaemic control may require analyses to apply varying cut points with respect to age, however this does introduce an additional element of arbitrariness with respect to which age cut-offs are used.

### Pathology testing data provides a limited indicator of quality of diabetes care

The breadth and depth of best-practice diabetes care cannot be measured by focusing solely on pathology-based tests. There are a number of aspects of quality diabetes care, which although important, cannot be measured within the scope and focus of this study

### Participation rates

Poor participation rates are common in GP research [39,40]. The study will implement a range of evidence-based strategies to increase participation. It should be noted that because the outcome measures are population based, even if participation is poor, the study will provide a ‘real world’ indication of the impact of these sorts of methods, given that the intervention components reflect best practice in CME.

## Conclusions

Despite the identified methodological challenges, the study data are likely to provide valuable information regarding monitoring and improvement of healthcare using ongoing administrative data sets at a population level. The study also has potentially wide-reaching applications for evaluating guideline adoption free from bias associated with provider and participant non-consent.

## Abbreviations

CME: Continuing medical education; NHMRC: National Health and Medical Research Council; ALM: Active learning module; RACGP: The Royal Australian College of General Practitioners; QI: Quality improvement; CPD: Continuing professional development; GP: General practitioner; NSW: New South Wales.

## Competing interests

The authors declare that they have no competing interests.

## Authors’ contributions

All authors contributed to trial design, trial coordination, and manuscript preparation. PM oversaw statistical aspects of the protocol. All authors read and approved the final manuscript.
